# Current chemotherapy strategies for adults with IDH-wildtype glioblastoma

**DOI:** 10.3389/fonc.2024.1438905

**Published:** 2024-07-19

**Authors:** Jing Bao, Rui Sun, Zhenjiang Pan, Shepeng Wei

**Affiliations:** Shidong Hospital, University of Shanghai for Science and Technology, Shanghai, China

**Keywords:** glioblastoma, chemotherapy, temozolomide, lomustine, MGMT

## Abstract

**Introduction:**

Glioblastoma, despite advancements in molecular evolution, remains incurable and has low survival rates. Currently, two of the most commonly used chemotherapy regimens are temozolomide and CCNU. This review aims to provide a comprehensive analysis of the current status of chemotherapy strategies for GBM.

**Methods:**

We reviewed the published literature describing the chemotherapy regimen differences in system treatment of GBM reported in the last ten years and summarised the available information that may reveal the latest changes in chemotherapy.

**Results:**

In patients with adequate functioning, temozolomide and radiation are the primary treatments for newly diagnosed GBM. We recommend postoperative radiation therapy with concurrent and adjuvant temozolomide for patients with MGMT-methylated GBM who are less than 70 years old. Combining temozolomide and lomustine with radiation therapy may be an option for younger, fit patients, but efficacy data is inconclusive. For patients with unknown MGMT methylation status, radiation therapy combined with temozolomide remains the standard of care. We recommend hypofractionated radiation and concurrent temozolomide treatment for elderly patients over 70 years old who have satisfactory performance and no significant underlying health conditions. We should tailor treatment choices to each patient’s personal preferences, previous treatments, function, quality of life, and overall care objectives.

**Conclusion:**

Radiation therapy, along with temozolomide, is still the standard of care for most people with MGMT-unmethylated GBMs because there aren’t any better options, and it’s generally safe and well-tolerated. These patients have a lower overall survival rate and less benefit from temozolomide, but there are no better alternatives. Clinical trial participation is encouraged.

## 1 Introduction

Glioblastoma (GBM) is a type of brain tumour that is believed to originate from neuroglial stem cells or their progenitors in the subventricular zone. It is classified as a subtype of adult diffuse glioma and is located in the primary central nervous system (CNS) (1–3). The incidence of GBM increases after the age of 40 and peaks in adults aged 75 to 84 years (4, 5).

Actually, GBM is the most prevalent malignant primary brain tumour, with a median survival rate of under 2 years. The median overall survival for IDH-wild-type GBM patients is between 12 and 21 months, with only approximately 7% of patients surviving for 5 years (6, 7).

**Figure 1:**
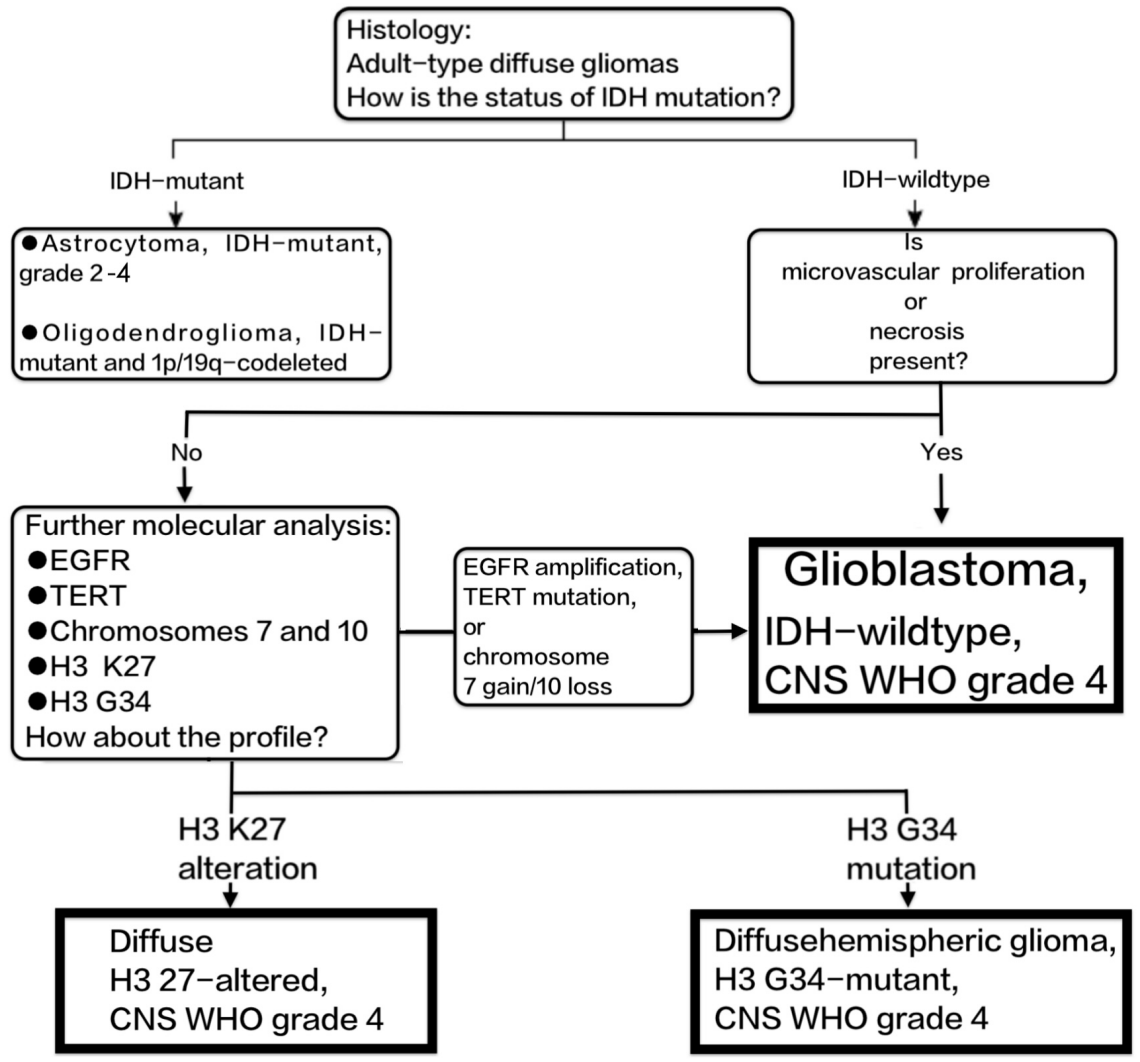
Shows the diagnostic process of GBM according to the 2021 revision of the WHO classification of CNS tumours.

Histopathologically, GBMs are characterised by pleomorphism, high cellularity, diffuse infiltration, mitotic activity, and either microvascular necrosis, proliferation, or both. At the molecular level, GBMs are characterised by the absence of mutations in IDH1/2, H3 K27M, and H3 G34, as per their definition. In diffuse gliomas that do not have mutations in the IDH and H3 genes, the presence of either microvascular proliferation or necrosis is enough to diagnose GBM as grade 4. Tumours that have epidermal growth factor receptor (EGFR) amplification, telomerase reverse transcriptase (TERT) promoter mutation, or concurrent chromosome 7 gain/chromosome 10 loss exhibit a clinical course similar to that of GBM (8, 9). These tumours are now classified as IDH-wildtype GBM according to the 2021 WHO revision (3). The diagnosis of GBM in adults is shown in **Figure 1**.

Consequently, GBM now specifically denotes the most severe type of isocitrate dehydrogenase (IDH)-wild-type diffuse adult-type astrocytoma. The prognosis of this condition is determined by the methylation status of the MGM promoter.

Although there has been progress in comprehending the molecular evolution of GBM, the disease remains incurable and is associated with low survival rates. The conventional multimodal treatments of surgically removing as much of the tumour as possible and using radiation therapy (RT), along with the simultaneous and subsequent use of temozolomide (TMZ), continue to be the mainstay of treatment. Clinical trials are ongoing to further explore innovative strategies, such as targeted agents and immunotherapy. This review aims to provide a comprehensive analysis of the current status of chemotherapy strategies for GBM.

## 2 Temozolomide: a classic old medicine

Temozolomide, an oral alkylating agent, is dosed according to body surface area (BSA). During radiation treatment, 75 mg/m^2^ of temozolomide is administered daily (seven days per week). Temozolomide is taken on an empty stomach, at least two hours after the previous meal.

Some clinicians recommend timing weekday doses of temozolomide one hour before radiation therapy to maximise synergy. Other clinicians have patients take their medication at the same time every day, either first thing in the morning before breakfast or at bedtime, two or more hours after dinner.

Complete blood counts (CBCs) must be done every week during the concurrent phase for monitoring. Platelets should not be given if they drop below 100,000/microL, or ANC should not be given if it drops below 1500/microL, because counts can drop quickly. After that, monitoring may need to happen more often until nadirs are found.

Temozolomide is usually given at a dose of 150 mg/m^2^ every day for five days out of a 28-day cycle. The first post-radiation cycle starts four weeks after the last day of radiation therapy. After the first six cycles, the dose is 200 mg/m^2^ if the blood counts are good. On days 22 and 29 of each cycle, a CBC should be done, along with a basic metabolic panel and liver function tests once a month. This is to check for toxicity and help with dose adjustments if needed. Clinicians should look at the temozolomide product label to see how to change the dose to avoid hematologic toxicity.

It is recommended to treat patients who have received standard concurrent and adjuvant temozolomide with six cycles of monthly adjuvant temozolomide instead of a longer treatment course.

Alternative temozolomide schedules have not demonstrated superior efficacy compared to the conventional postradiation schedule of 5 days every 28 days in the adjuvant setting (10–12).

During radiation with concurrent temozolomide, patients with additional risk factors for opportunistic infections (such as lymphopenia or the use of glucocorticoids) should be given antimicrobial prophylaxis to prevent pneumocystis pneumonia. Given the low risk of pneumocystis, the risks of prophylaxis may be greater than the benefits for other patients.

## 3 GBMs, age ≤70 years

### 3.1 MGMT-methylated GBMs

A European Organisation for Research and Treatment of Cancer/National Cancer Institute of Canada (EORTC/NCIC) open-label trial in 573 adults with GBM randomised them to receive involved-field radiation therapy alone or radiation plus concurrent daily temozolomide followed by up to six monthly cycles of adjuvant temozolomide (6). After five years, temozolomide improved median overall survival compared to radiation alone (14.6 versus 12.1 months, HR 0.63, 95% CI 0.53-0.75) (6). Temozolomide improved survival at two years (27 versus 11%) and five years (10 versus 2%) (13). Temozolomide caused more grade 3 or 4 hematologic toxicity, mostly thrombocytopenia (12%) and lymphopenia (7%) but maintained health-related quality of life (6, 14). All patient subsets, including those over 60 and those with poor prognostic factors, benefited from adjuvant temozolomide (6, 13). A smaller phase II trial in GBM patients yielded similar results (15).

In a retrospective analysis of 206 EORTC/NCIC trial patients, MGMT promoter methylation was a major prognostic factor for improved survival and chemotherapy benefit (16). People with MGMT methylation (45% of cases) had twice as much two-year overall survival with temozolomide as with radiation alone (46% vs. 23%); their median overall survival was 21.7 months vs. 15.3 months, with an HR of 0.45 and a 95% confidence interval of 0.32-0.61 (16). Those without MGMT methylation showed a non-significant survival difference (two-year survival: 14% vs. <2%; median overall survival: 12.7 vs. 11.8 months, HR 0.69, 95% CI 0.47-1.02).

Later trials in MGMT-methylated GBM have tried to improve radiation plus temozolomide. CeTeG/NOA-09 screened >650 patients to enrol 141 patients aged 18 to 70 with MGMT-methylated GBM and randomly assigned them to a combined lomustine/temozolomide regimen during and after radiation therapy (up to six 42-day cycles of lomustine 100 mg/m^2^ on day 1 and temozolomide 100 mg/m^2^ on days 2 to 6) or standard therapy (17). Centre-stratified randomization, A modified intent-to-treat (mITT) analysis included 129 patients after 12 randomised patients (six in each arm) dropped out before therapy. Sex distribution and other baseline prognostic factors differed statistically and numerically between treatment groups. Lomustine/temozolomide and standard temozolomide had similar median overall survival in mITT patients (37.9 vs. 31.4 months, HR 0.90, 95% CI 0.58–1.41). Using inverse probability weights, an adjusted mITT analysis of these 129 patients found a nonsignificant trend towards improved survival in the lomustine/temozolomide arm (HR 0.74, 95% CI 0.47–1.17) but no difference in progression-free survival. This matched analysis (n = 109), which excluded 32 randomised patients, showed that the combination arm had better overall survival (48.1 versus 31.4 months, HR 0.60, 95% CI 0.35–1.03) and similar progression-free survival (16.7 months in both groups). Grade 3 or 4 hematologic toxicity (36 vs. 29%) and nausea (30 vs. 19%) were more common with combination therapy. Lomustine/temozolomide completed 39 percent of six chemotherapy cycles, while standard temozolomide completed 60 percent.

These findings suggest that lomustine/temozolomide combination therapy may improve survival in younger, fit MGMT-methylated GBM patients compared to standard temozolomide. The trial’s small size and the exclusion of a large number of randomised patients in the prespecified analyses reduce confidence in combination therapy’s superior efficacy. Combination therapy has higher nausea and hematologic toxicity risks, so some patients and clinicians may prefer standard temozolomide until more is known.

So, we suggest that people younger than 70 who have been newly diagnosed with MGMT-methylated GBM get temozolomide and radiation therapy at the same time, followed by temozolomide every month. Younger, fit patients with MGMT-methylated tumours may benefit from temozolomide and lomustine combined with radiation therapy, but efficacy is inconclusive and toxicity may be higher.

### 3.2 MGMT-unmethylated GBMs

Patients with MGMT-unmethylated tumours may benefit from clinical trials due to their poor prognosis and response to standard treatments. To treat MGMT-unmethylated GBM, we recommend temozolomide and radiation outside of clinical trials, based on the EORTC/NCIC trial, where MGMT status was not known (6, 13).

Patients with MGMT-unmethylated tumours have lower overall survival and less benefit from temozolomide compared to those with methylated tumours. In a retrospective analysis of 206 EORTC/NCIC trial patients with MGMT-unmethylated tumours (n = 114), adding temozolomide to radiation therapy resulted in a non-significant survival advantage (two-year survival 15 versus 2%; median overall survival 12.7 versus 11.8 months, HR 0.69, 95% CI 0.47-1.02) (16).

Investigations are underway for alternatives to temozolomide in MGMT-unmethylated tumour patients. For example, a phase II study of 182 people who had just been diagnosed with MGMT-unmethylated GBM looked at the effects of bevacizumab during radiation, bevacizumab plus irinotecan, and radiation with temozolomide given at the same time or afterward (18). Similar to unselected GBM trials, bevacizumab improved six-month progression-free survival (79 versus 43 percent) but not median overall survival (16.6 versus 17.5 months) or quality-of-life. Bevacizumab was given to two-thirds of temozolomide patients at progression. In a trial comparing radiation and nivolumab to radiation and temozolomide, the nivolumab arm had lower survival rates (median overall survival 13.4 vs. 14.9 months, HR 1.31, 95% CI 1.09–1.58) (19).

### 3.3 MGMT status unknown

Due to insufficient tissue, MGMT methylation assays may fail in a significant minority of patients, especially those who undergo stereotactic biopsy. We recommend using temozolomide with radiation therapy for patients who are candidates for standard therapy (age ≤70 years, good functional status) and whose MGMT status is unknown at the time of decision-making. The rationale for temozolomide is the expected clinically significant survival improvement for 30–40% of patients with MGMT-methylated tumours, the lack of better alternatives for unmethylated tumours, and its relative safety and tolerability.

## 4 GBMs, age >70 years

### 4.1 Radiation with concurrent and adjuvant temozolomide

For individuals over 70 years old who exhibit good performance status (e.g., Karnofsky Performance Status [KPS] ≥70), hypofractionated radiation (e.g., 40 Gy in 15 fractions) in conjunction with adjuvant and concurrent temozolomide is recommended. However, in older patients compared to younger patients, the potential for improved survival with the addition of chemotherapy is more closely balanced with the risks of toxicity. Individuals with specific concerns regarding side effects may logically opt for monotherapy.

The results of a randomised trial of hypofractionated radiation (40 Gy in 15 fractions) with or without concurrent and adjuvant temozolomide provide support for combined-modality therapy in older patients with newly diagnosed GBM (20). Patients with an Eastern Cooperative Oncology Group (ECOG) score of 0 to 2 who were 65 years of age or older were eligible. 562 patients with a median age of 73 years (range 65 to 90) were included in the trial. Results include the following:

- When temozolomide was added to radiation, the survival rate was higher than when radiation was used alone (9.3 versus 7.6 months, hazard ratio [HR] 0.67, 95% CI 0.56-0.80). Additionally, progression-free survival increased (5.3 versus 3.9 months).
- MGMT was examined in 354 patients. In 165 patients with MGMT-methylated tumours, adding temozolomide increased overall survival by nearly six months (13.5 vs. 7.7 months, HR 0.53, 95% CI.38–0.73). Despite a smaller effect (10 versus 7.9 months, HR 0.75, 95% CI 0.56–1.01), temozolomide improved survival in patients with MGMT unmethylated tumours (n = 189).
- Functional domain quality-of-life outcomes were similar. More nausea, vomiting, constipation, and hematologic toxicity (grade 3 or 4)—thrombocytopenia (11 versus 0.4 percent), neutropenia (8 versus 1 percent), and lymphopenia (27 versus 10 percent)—were seen in the combined therapy.

Extra supporting information comes from observational studies involving older adults, which might have bias related to selection (21, 22). The median overall survival was 13 months in a pooled analysis of four phase II trials, including older patients (>65 years old) with newly diagnosed GBM treated with concurrent and adjuvant temozolomide plus standard or short-course radiation therapy. This result compared favourably with outcomes in younger patients (21). The prevalence of grade 3 or 4 toxicity ranged from 8 to 46%.

Although the patient population for the seminal European Organisation for Research and Treatment of Cancer/National Cancer Institute of Canada Clinical Trials Group (EORTC/NCIC) trial was restricted to individuals aged 18 to 70, a breakdown of outcomes by age was incorporated into the five-year analysis of results (13). Thirty percent (170) of the 573 patients in that study were between the ages of 61 and 70. The combined-modality approach’s median overall survival for this older patient subset was comparable to that of radiation therapy alone (median 10.9 versus 11.8 months).

Adjuvant temozolomide significantly increased overall survival compared to radiation therapy alone, resulting in more long-term survivors (22% versus 6% at two years and 7% versus 0% at five years, HR 0.7, 95% CI 0.5-0.97).

In older patients, at least one retrospective study found that combined therapy had worse outcomes than radiation alone (23), and other studies found that the benefit of adding temozolomide decreased with age (24), particularly in those with MGMT unmethylated tumours (25).

### 4.2 Temozolomide alone

Emerging data suggests that temozolomide chemotherapy may be a viable alternative to radiation therapy for older patients with MGMT-methylated tumours who cannot benefit from a combined-modality approach due to poor functional status or significant comorbidity.

Two randomised trials in older patients provide partially overlapping data on the best approach for this population, specifically the role of temozolomide alone as an alternative to radiation. Both the Methusalem (NOA-08) trial (26) and the Nordic Clinical Brain Tumour Study Group trial (27) compared initial chemotherapy as monotherapy to initial radiation alone. Neither trial had a combined temozolomide-radiation arm.

These trials indicate that both hypofractionated radiation and temozolomide are viable treatment options for older patients. However, temozolomide is more effective in patients with MGMT-methylated tumours than in those with unmethylated tumours.

**Figure 2:**
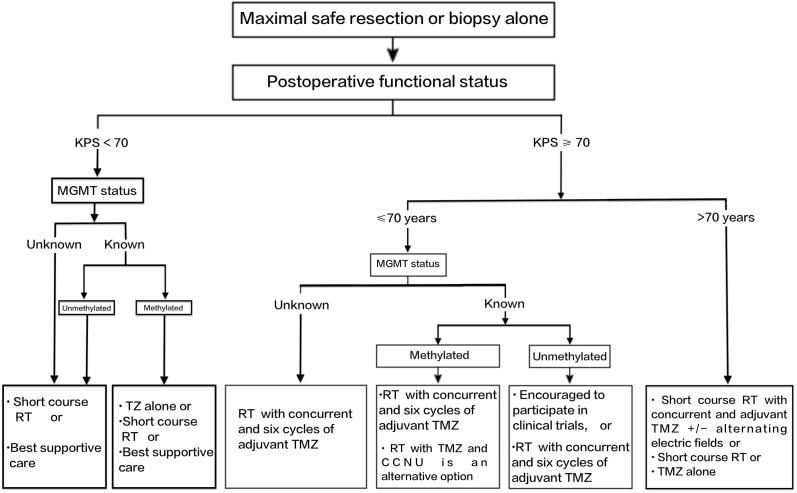
Initial approach to chemotherapy in GBM patients. TMZ, temozolomide; CCNU, lomustine; Rt, radiation therapy.

**Table 1** summarises the initial chemotherapy drug regimen for GBM. In **Figure 2**, the clinical approach for the initial selection of chemotherapy drugs for GBM is illustrated.

**Table 1:** Initial chemotherapy strategies for adults with IDH-wildtype glioblastoma.

KPS≥70	
Age ≤70 years	
MGMT -methylated	•Daily TMZ with standard Rt followed by ≤ 6 cycles of monthly TMZ •TMZ + CCNU in combination with Rt
MGMT -unmethylated	•Encouraged to participate in clinical trials. •Daily TMZ with standard Rt followed by ≤ 6 cycles of monthly TMZ
MGMT status unknown	•Daily TMZ with standard Rt followed by ≤ 6 cycles of monthly TMZ
Age >70 years	
no matter what MGMT status	•Daily TMZ with short-course Rt followed by ≤ 6 cycles of monthly TMZ
KPS<70	
•Rt alone •TMZ chemotherapy alone, particularly in patients with MGMT methylated tumors. •Best surpportive care	

TMZ, temozolomide; CCNU, lomustine; Rt, radiation therapy.

## 5 Recurrent GBM

The systemic agents most frequently employed in the treatment of recurrent GBM include bevacizumab, nitrosoureas, and temozolomide rechallenge (as the majority of patients would have already received temozolomide as part of their initial therapy). Whenever feasible, it is preferable for patients with a satisfactory performance status to engage in a research clinical study.

Prospective studies in patients with recurrent GBM have shown that using only lomustine as a treatment results in a response rate of 9 to 14 percent and a median progression-free survival of 1.5 to 2.7 months (28–30). The typical initial dosage for monotherapy is 110 mg/m^2^, with a maximum limit of 200 mg. Approximately 50 percent of individuals experience grade 3 or higher hematologic toxicity.

The largest study, known as RESCUE, examined the effects of continuous daily administration of temozolomide at a dosage of 50 mg/m^2^/day for a maximum duration of one year in a group of 120 patients (31). The six-month progression-free survival rate for patients with GBM varied between 15 and 29 percent, depending on whether progression occurred during or after the initial adjuvant temozolomide treatment. The patients who underwent a rechallenge after completing an adjuvant regimen exhibited the highest likelihood of responding.

A randomised phase II trial was conducted to compare the efficacy of two different dosing regimens of temozolomide (150 mg/m^2^/day one week on, one week off, or 100 mg/m^2^/day three weeks on, one week off) in patients with recurrent GBM. However, the trial had to be stopped prematurely due to a lack of funding. Despite the early termination, both dosing regimens showed similar performance (32). MGMT status was the primary determinant of effectiveness. The six-month progression-free survival was significantly higher in patients with MGMT-methylated tumours compared to those with unmethylated tumours, regardless of the dosing regimen. The survival rate was 40 percent for patients with MGMT methylated tumours, while it was only 7 percent for patients with unmethylated tumours.

Similar to other medications, the administration of high-dose temozolomide in patients who have not responded well to bevacizumab-containing treatment plans is linked to a low rate of positive response and overall survival (33–35).

## 6 Treatment duration

Based on the design of the original study that led to the acceptance of temozolomide as the standard of care, up to six cycles of postradiation temozolomide are recommended (6).

The results of a phase II randomised trial, in which 159 glioblastoma patients who had not progressed after six cycles of adjuvant temozolomide were randomly assigned to stop temozolomide (control) or continue temozolomide for up to 12 cycles overall, provide evidence in favour of this approach (36). The groups’ progression-free survival was comparable after a median follow-up of 33 months, and the extended temozolomide group’s overall survival was not significantly worse (hazard ratio [HR] 1.3, 95% CI 0.90-1.88).

Comparable findings have been drawn from observational studies (37, 38). A retrospective study of 624 patients in four randomised trials found that receiving more than six cycles of adjuvant temozolomide was associated with improved progression-free survival, particularly in patients with MGMT-methylated tumours (HR 0.65, 95% CI 0.50-0.85). However, there was no difference in overall survival (HR 0.92, 95% CI 0.71-1.19), even in the MGMT-methylated subgroup (HR 0.89, 95% CI 0.63-1.26) (38).

## 7 Conclusion

Looking at the chemotherapy regimens for GBM, only two drugs, TMZ and CCNU, are currently in use (**Table 1**). Temozolomide and radiation are the primary components of the initial treatment for newly diagnosed GBM in patients who have a sufficient level of functioning. For patients with MGMT-methylated GBM ≤70 years old, postoperative radiation therapy with concurrent and adjuvant temozolomide is recommended. Combining temozolomide and lomustine with radiation therapy may be an option for younger, fit patients, but efficacy data is inconclusive and toxicity may be higher.

For patients with MGMT-unmethylated GBM ≤70 years old, postoperative radiation therapy with concurrent and adjuvant temozolomide is recommended. These patients have lower overall survival and less benefit from temozolomide, but there are no better alternatives. Clinical trial participation is encouraged.

For patients with unknown MGMT methylation status (≤70 years), based on the clinically meaningful improvement in survival that is anticipated from temozolomide for the 30 to 40 percent of patients who are predicted to have an MGMT-methylated tumour, the absence of better alternatives for MGMT-unmethylated tumours, and the relative safety and tolerability of temozolomide, radiation therapy combined with temozolomide continues to be the standard of care.

In the case of elderly patients (>70 years old) who have a satisfactory performance status and no significant underlying health conditions, it is recommended to use hypofractionated radiation (such as 40 Gy delivered in 15 fractions) along with concurrent and adjuvant temozolomide treatment (administered in six monthly cycles) instead of a single treatment modality. With advancing age, the potential for improved survival more closely balances the risks of toxicity associated with combination therapy. However, patients who have significant concerns about side effects may justifiably opt for single-modality therapy.

In the case of elderly patients (>70 years old) who are not candidates for a combined-modality approach because of poor functional status or significant comorbidity, the MGMT methylation status of the tumour is useful for decision-making. In cases where patients have tumours with MGMT methylated, temozolomide rather than radiation is recommended.

Although a combined-modality approach is employed, the majority of patients ultimately experience a relapse. Managing patients with recurrent or progressive high-grade gliomas is challenging, and there is no evidence that active reintervention extends survival. At this point, treatment choices should be tailored to each patient, considering their personal preferences, previous treatments, ability to function, quality of life, and overall care objectives.

Patients who have experienced a relapse a few months after the completion of adjuvant temozolomide treatment and whose tumours contain a methylated MGMT promoter may be the most suitable candidates for rechallenge with temozolomide.

## Author contributions

JB: Writing – original draft, Writing – review & editing. RS: Writing – original draft, Writing – review & editing, Investigation, Methodology. ZP: Writing – original draft, Writing – review & editing. SW: Writing – original draft, Writing – review & editing, Conceptualization, Methodology, Resources.

## Author disclaimer

All claims expressed in this article are solely those of the authors and do not necessarily represent those of their affiliated organizations, or those of the publisher, the editors and the reviewers. Any product that may be evaluated in this article, or claim that may be made by its manufacturer, is not guaranteed or endorsed by the publisher.

The opinions stated in this article are exclusively those of the authors and may not necessarily reflect the views of their associated organisations, or those of the publisher, the editors, and the reviewers. The publisher does not guarantee or endorse any product mentioned in this article or any claims made by its manufacturer.
